# On Croton Oil

**Published:** 1823-11

**Authors:** 


### Art. II.—*On Croton Oil.* By Dr. Kinglake.

This oil is obtained by expression from the seeds of a plant termed *croton tiglium*, a native of the island of Ceylon, and found in China, the Molucca islands, and in various parts of the East Indies. The inhabitants of the different countries in which it spontaneously grows have immemorially regarded every part of the plant, root, stalk, and leaves, as well as the seed, as violently cathartic, and have occasionally used them in that intention in cases of obstinate constipation. The acrid principle, on which the cathartic power of the plant depends, resides in its resinous part, which forms a large portion of its general bulk. This highly stimulant material pervades its whole structure, but seems to be chiefly concentrated in the seminal product.

From the seeds of this plant the croton oil in modern use is obtained. It is perhaps the most commodious and efficient form in which the medicinal powers of this vegetative production could be administered. The seeds have been long known in Europe, under the name of *grana Molucca* and *grana tiglia*, and have been given, either dried and reduced to powder, or in the moist state conjoined with honey, in the form of pill, to the extent of one grain, in cases requiring prompt and active excitement of the peristaltic power of the intestines.

Nearly one-half of the whole bulk of croton oil has been ascertained to consist of an acrid principle, which, if undiluted, and not in some measure obtunded by the oily vehicle forming the other part, would probably be much too subtle and impressive in its influence on vital organization, to admit of being safely employed. It would, perhaps, by a divellent agency, instantly decompose the organic structure from which vital power emanates, and, by this destructive operation, prove the most active of all known poisons. It would therefore not be desirable to detach this extremely acrid substance from the oily envelop by which it is prevented from exerting the full extent of its virulent power.

All cathartic substances, in common with other descriptions of medicinal stimulants, derive their efficient properties from an arrangement of matter somewhat similar to that which is inherent in croton oil. The main difference consists in the degree of concentration in which the active principle exists. Substances operating moderately on the peristaltic power of the bowels have the active principle so widely diffused in the texture in which it resides, as not to allow of much union or aggregation of power. Elaterium, colocynth, aloes, gamboge, and buckthorn, approach to the condensed strength of croton oil, but are still very inferior to it in effective power.

It is certainly a desideratum to have an agent like croton oil, to which a last resort may be made in cases of great emergency, with a prospect of obtaining the relief that could not be effected by any other known means, and without which death would inevitably ensue. Valuable as this resource may be in cases of insuperable difficulty by the usual modes of affording aid, yet due caution should be observed in exhibiting an agent that would seem, by its stupendous powers, to be fraught with the issues of life and death. The power that could be rapidly efficient in circumstances in which no assistance could be derived from means ordinarily found to be fully adequate to all that could be desired, exerts an energy of action that may be either salutary or destructive. If it should disorganize, it cannot prove remedial, and the limit between such an effect and the desired relief is so precariously uncertain as to render the result extremely ambiguous. The indication, then, for the use of such a potent agent as croton oil can only be furnished by ample proof of the insufficiency of those medicines which generally produce the required effect without any attending risk, or cause for apprehending any disastrous influence.

The reputation of a third or half part of a drop of croton oil being a sufficient dose where the most powerful cathartic medicines had been previously resisted, always suggested to my apprehension that its agency would be of a nature rather to extinguish the sinking energies of life, than to excite them to salutary action. It is probable that the torpid and much exhausted state of vital power in the cases in which croton oil has been employed, is the negative condition that ensures its beneficial effect, and protects against its pernicious operation.

Would croton oil act safely on an unembarrassed and unexhausted state of vital power, such as occurs in the villous coat of the stomach and intestines in common instances of constipation? The pain that is usually endured in intestinal obstruction, and the unavailingly stimulant remedies commonly given on those occasions, reduce vital energy to a low ebb, which probably enables it to bear the high exciting agency of croton oil, not only with impunity, but with the cathartic effect that procures the desired relief. If croton oil, therefore, like spirit of turpentine, be had recourse to in cases of intestinal obstruction after other powerful means have failed, and sufficiently early to obtain evacuations before gangrene may be induced, it promises to prove a most important addition to the list of efficient medicines in one of the most deplorable ailments incident to human nature.

An opportunity has been very lately afforded me of subjecting the cathartic power of croton oil to the test of experiment, in two instances in the same patient. On the first occasion, hepatic derangement occurred, attended with a severity of pain that denoted a distended and obstructed state of the biliary ducts by concreted or inspissated bile. The first passages were soon involved in the disturbance, inducing vomiting and a torpid condition of the peristaltic power of the intestines, that resisted, during upwards of two days and nights, the most efficient remedies usually employed in these cases. Terebinthinate clysters, mercurial, aloetic, and other cathartic medicines, were exhibited in large doses at short intervals, but without making the slightest impression on the existing constipation. The general strength began at length to give way, and symptoms of destructive exhaustion, or rather of gangrene, became apparent, and placed the patient's life in the most imminent danger. Under these circumstances, the attending surgeon, Mr. Lidden, of this town, at my suggestion, administered croton oil, in the dose of one drop conjoined with three grains of extract of colocynth, in the form of pill. It was proposed to repeat the dose every hour, until some decisive effect should be produced. The first dose was retained, and excited rumbling in the bowels, but no evacuation within the hour. A second dose was then given, which operated within half an hour most copiously. Neither nausea, pain, nor any sensible inconvenience, attended its energetic action. It proved the happy means of removing a very large quantity of dark-looking, fetid, and scybalous substances, from the bowels. The relief afforded was immediate and complete; all pain, sickness, and vomiting, speedily subsided. Diluting and dietetic articles were afterwards retained on the stomach, and the patient remained for about three or four days free from complaint. After the lapse of that time, hepatic pain and inactive bowels recurred. Common remedies were again found to be unavailing; the croton oil was therefore resumed, in a similar dose to that in which it had been before given. The first drop in this instance produced very fully the desired effect, within half an hour of its being taken. Since that time the patient gradually recovered, the bowels for the most part resuming their natural action, and admitting of being occasionally moved by slight aperient medicines, such as castor oil, magnesia, or rhubarb.

Were these instances of the efficacy of croton oil characteristic of the uniform power of the remedy, it would deserve to be hailed as a medicine, in such circumstances, of inappreciable worth. To afford all the aid that could be desired in the last extremity of the most hopeless constipation, is an extent of relief that cannot be sufficiently estimated. It is precisely the sort of assistance that is often required, and without which death must inevitably ensue.

It has been my happy lot to experience the high gratification of rescuing two patients from imminent peril of death, by the cathartic power of spirit of turpentine. Like croton oil, the spirit of turpentine restrained the inverted action of the stomach and bowels, that caused every thing that was swallowed previously to its being taken to be speedily rejected by vomiting. This desirable and auxiliary contribution to its final efficacy seems to be similarly effected by both articles, by strongly exciting and energizing the peristaltic power, so as to enable it to exert sufficient contractile force to transmit and expel from the intestines the obstructed substances that could not be removed by any other known agents.

Croton oil, from the smallness of the dose in which it is efficient, and the flavourless concealment with which it may be given, in the form of either pill or bolus, is preferable to spirit of turpentine, if its remedial effects in obstinate constipation be fully equal to those of that article. It is, however, an advantage to have two such potent agents at hand, supplying an alternative, if either should fail in procuring the necessary evacuation.

Croton oil seems to act expeditiously and commodiously in the form of pill. It may be made into that shape with either crumb of bread, honey, conserve of roses, extract of hyoscyamus, that of colocynth, jalap, or any other similar vehicle. As the concentrated strength of the oil in the dose of one drop is well borne on the stomach, without inducing either a sense of excessive heat, sickness, or pain, it is probably the most efficient and advantageous form of administering it. Were it to be more diluted by being mixed with an aqueous or any other fluid, its power would, perhaps, be too much attenuated to enable it to act with the promptitude that may prevent the nauseating and other inconveniences that would be likely to result from a slower and less decided exertion of its cathartic power. Swallowed as an oleo saccharum, by gradual solution, it has operated speedily and without molestation.

Although it may be expedient first to try, in all instances of obstructed bowels, the effect of common purgatives, yet it would not be advisable to delay the more efficient aid that may be derived from either croton oil or spirit of turpentine for an indefinite period, lest gangrene should supervene, which would render the evacuating power of either of these remedies unavailing. In hernial obstruction of the intestinal canal, the attempt to replace the prolapsed portion of the bowel within its natural cavity is too often fruitlessly protracted, until sphacelus has been produced, after which the operation of dilating the abdominal ring by incision can afford no benefit. In these cases, the effectual relief derivable from a seasonable use of the knife should not be postponed until irremediable mischief has been incurred; nor should the powerful influence of croton oil and spirit of turpentine, when necessary, be deferred until the vital power of the intestines be too much sunk and disorganized to admit of being salutarily excited.

*Taunton; June 6th,* 1823.

P.S.—Since writing the above observations on the medicinal effects of croton oil, a case of a very extraordinary and instructive description of obstructed intestines has fallen under my professional notice, in which that powerful cathartic ultimately succeeded. The patient was a female, aged about twenty-five, who had long been habitually subject to constipated bowels, arising from dyspeptic and other causes, inducing an atonic and torpid state of the alimentary canal. In the instance referred to, the customary costiveness became unyieldingly obstinate. The various cathartic medicines that are in general actively efficient were, during three days, unavailingly given. Clysters, likewise, of the most stimulating and powerful kind, were added to the medicinal attempt to produce on the peristaltic power a transmitting or purgative action. Croton oil, to the extent of one drop every hour, in three successive instances, had been fruitlessly taken. In common with all other cathartic remedies, each dose was rejected from the stomach within an hour after it was swallowed. At this period my attendance was desired in consultation, when it was determined that the croton oil, as holding a decided precedence as a rapid and powerful purgative, should be repeated every hour until either the desired effect should be produced, or an objection to its continuance, on the score of its being insufferably stimulating, should arise.

Pursuant to this arrangement, two additional doses were given of two drops each, at intervals of one hour; but the nausea, vomiting, and pain, that ensued, having been attributed to the inefficiency of the remedy, the patient could not be induced to persist in any further trial of its power. Three additional days, making altogether six, elapsed without evacuation from the bowels having been procured, during which time two ounces of rectified spirit of turpentine, and other powerful cathartic medicines, were administered, and the patient also diluted copiously with a saturated solution of phosphate of soda. At this period, every article that was swallowed was speedily rejected with great violence, accompanied with a sense of the most acute spasmodic pain about the obstructed portion of the bowels. There was but little or no abdominal tension, and external pressure was borne without much inconvenience. The general arterial action was not much excited; the skin was open and steadily moderate in its temperature, and no chilliness or rigor occurred.

The patient appeared to be inexorably repugnant to all further medicinal attempts for relief, but was, however, at length prevailed on to swallow an ounce of quicksilver, which was done in two instances at intervals of one hour. A feeling of oppression followed this last remedy, but it did not at all pervade the obstructed part. This was the sixth day of the obstruction, without any relief having been effected. All medical attendance and endeavour to overcome the complaint were now absolutely prohibited; the persuasion of the patient, as well as the sympathy of surrounding friends, conceiving that any further attempt to relieve would not only be useless, but would aggravate the existing pain, and render the closing scene of life more afflicting. In this abandoned state, the patient resolutely continued until the twelfth day of the constipation, taking only a few drops of tincture of opium two or three times a-day, to lull pain and to smooth, as was expressed, the road to dissolution. Accident afforded me an opportunity, on the eighth day of the constipation, and on the second after all medical aid had been peremptorily refused, to contrive the introduction of four drops of croton oil in the dose of opiate medicine that was, by the patient's own desire, taken at intervals. This quantity of the oil produced a searching kind of excitement throughout the intestines, that occasioned a decided objection to even repeating the opiate, lest the uneasy sensation should be increased by it. During three days a sense of violent forcing was experienced in the bowels, accompanied with occasional vomiting. At length, on the fourth day of this propulsive sensation, the obstruction gave way, and copious discharges of scybalous, dark-coloured, and extremely fetid substances ensued. The evacuations continued without medicine, at short intervals, during the ensuing twenty-four hours. The previous vomiting, pain, and every other diseased feeling, soon subsided; the patient became decidedly convalescent, and has since regained her former state of health.

The practical reflection that suggests itself in this singularly wayward case, is that the cathartic power of the croton oil was not steadily applied and exerted after it was commenced. The vomiting that ensued, and the additional pain that it seemed to have occasioned, were no sufficient objection to assiduously persisting in its use, knowing that symptoms of active inflammation were not produced, and that, if the intestinal canal were mechanically pervious, the desired effect would possibly, sooner or later, result from its progressive influence. Intestinal obstruction from hernia, introsusception, and stricture obliterating the canal, oppose perhaps insuperable barriers to the powers, as well of croton oil, as of all other modes of medicinal aid; but the existence of such a state is always conjectural, and therefore can never be sufficiently evident to preclude the expediency of making attempts to overcome the obstacle to the utmost point of trial. If croton oil had been unremittedly given in suitable doses, and at proper intervals, when it was relinquished, the ultimate relief that was afforded by its desultory and unconnected use would probably have been produced at a much earlier period, and have exempted the patient from indescribable suffering, and the most imminent risk of a mortal issue. The four drops of the remedy that were by contrivance unconsciously conveyed into the first passages, through the medium of the opiate vehicle, succeeded in removing the obstruction after the lapse of four days from the time they were given. Had that dose been followed by another of an equal, or even less, power after a short interval, the eventual relief would most likely not have been so long delayed.

The case, on the whole, is highly interesting, in at once vindicating the cathartic power of croton oil from insufficiency, and demonstrating the error of ever abandoning obstructed bowels as irremediable, whilst the ability to swallow remains, and the evidence of existing gangrene is doubtful.

Another case of unyielding obstruction of the bowels was, so late as yesterday, placed under my professional direction, in which, after two days and nights had been consumed in unprofitably administering various powerful cathartic medicines at short intervals, one drop of croton oil, formed into a pill, with three grains of the compound extract of colocynth, removed the obstruction in the course of twenty minutes after it was swallowed. The direction was to repeat the dose every hour until the bowels should be freely moved; the discharge, therefore, having not been, in the estimation of the patient's attendants, sufficiently copious within an hour of the time of giving the first pill, a second, containing another drop of the oil, was taken, which was speedily followed by profuse evacuations. The relief derived from the cathartic operation of the croton oil was early, and, as usual, all that could be reasonably desired. No additional pain, that the patient could distinguish from that which was induced by the morbid obstruction, was experienced from the influence of the oil; the previous vomiting ceased, the tension of the abdomen became less, and the pain which had been felt on pressure was greatly diminished.

The patient was a house-painter, and had been occupied in that pursuit when the obstruction began, and became painful: it may therefore be regarded as an instance of colica pictonum, and it appeared to be a case of a very severe and portentous description. Previously to taking the oil, all medicine, in common with every domestic article that was swallowed, was speedily rejected by vomiting. The oil was retained, and, by its peculiar efficiency on the contractile or peristaltic power of the intestines, rapidly removed the existing obstruction, and thereby obviated the fatal termination which imminently threatened the case, if seasonable aid had not been obtained.

*Taunton; August 30th*, 1823.

